# Dexmedetomidine attenuates spinal cord ischemia–reperfusion injury through both anti-inflammation and anti-apoptosis mechanisms in rabbits

**DOI:** 10.1186/s12967-018-1583-7

**Published:** 2018-07-21

**Authors:** Zhixiang Sun, Tianyun Zhao, Shaojun Lv, Ying Gao, Joe Masters, Hao Weng

**Affiliations:** 1 Department of Anesthesiology, Shanghai Fengxian District Central Hospital, Southern Medical University, Shanghai, People’s Republic of China; 2 Department of Anesthesiology, Guangzhou Women and Children’s Medical Center, Guangzhou Medical University, Guangzhou, People’s Republic of China; 3 Department of Anesthesiology, The First Affiliated Hospital of Guangzhou University of Chinese Medicine, Guangzhou, People’s Republic of China; 4 Anaesthetics, Pain Medicine and Intensive Care, Imperial College London, Chelsea and Westminster Hospital, London, UK; 5 Department of Anesthesiology, Shanghai Fengxian District Central Hospital, Southern Medical University, Fengxian District, Shanghai Nanfeng Road on the 6600th, Shanghai, People’s Republic of China

**Keywords:** Dexmedetomidine, Spinal cord ischemia–reperfusion injury, Neuroprotection, Apoptosis, Inflammatory responses

## Abstract

**Background:**

Dexmedetomidine (Dex) can improve neuronal viability and protect the spinal cord from ischemia–reperfusion (I/R) injury, but the underlying mechanisms are not fully understood. This study investigated the effects of dexmedetomidine on the toll-like receptor 4 (TLR4)-mediated nuclear factor κB (NF-κB) inflammatory system and caspase-3 dependent apoptosis induced by spinal cord ischemia–reperfusion injury.

**Methods:**

Twenty-four rabbits were divided into three groups: I/R, Dex (10 µg/kg/h prior to ischemia until reperfusion), and Sham. Abdominal aortic occlusion was carried out for 30 min in the I/R and Dex groups. Hindlimb motor function was assessed using the Tarlov scoring system for gait evaluation. Motor neuron survival and apoptosis in the ventral grey matter were assessed by haematoxylin–eosin staining and terminal deoxynucleotidyl transferase-mediated dUTP biotin nick end labelling staining. The expression and localisation of ionised calcium-binding adaptor molecule 1, TLR4, NF-κB and caspase-3 were assessed by immunoreactivity analysis. The levels of interleukin 1β and tumour necrosis factor α were assessed using enzyme-linked immunosorbent assays.

**Results:**

Perioperative treatment with dexmedetomidine was associated with a significant preservation of locomotor function following spinal cord ischemia–reperfusion injury with increased neuronal survival in the spinal cord compared to control. In addition, dexmedetomidine suppressed microglial activation, inhibited the TLR4-mediated NF-κB signalling pathway, and inhibited the caspase-3 dependent apoptosis.

**Conclusions:**

Dexmedetomidine confers neuroprotection against spinal cord ischemia–reperfusion injury through suppression of spinal cord inflammation and neuronal apoptosis. A reduction in microglial activation and inhibition of both the TLR4-mediated NF-κB signalling pathway and caspase-3 dependent apoptosis are implicated.

## Background

Spinal cord ischemia–reperfusion (I/R) injury is one of the most serious complications of major vascular surgery, and can lead to paraplegia after thoracoabdominal aortic aneurysm repair [1]. The mechanisms of spinal cord I/R injury are complex and include inflammation, apoptosis, excitatory amino acid toxicity, and calcium overload, all of which contribute to neuronal cell death. Spinal cord I/R injury occurs in up to one-fifth of high-risk patients [2]. Although there are some potential measures that have shown promise (such as controlled hypothermia and hyperbaric oxygen therapy) [3, 4], there is an urgent need to develop further neuroprotective strategies to target this devastating complication.

Dexmedetomidine (Dex), a selective α-2 adrenoceptor agonist, is a useful adjuvant to general anaesthesia which has sedative, anxiolytic, analgesic, and hypotensive properties. In recent years its potential organoprotective effects has become a major research direction [5]. In vitro and in vivo preclinical studies have widely demonstrated that Dex provides organoprotection in kidney, lung, brain, heart, and liver tissues by ameliorating I/R injury, inhibiting pro-inflammatory signalling pathways, and decreasing cell death [6–15]. There is also evidence that Dex can improve neuronal viability and preserve lower limb locomotor function in spinal cord I/R injury models [16, 17]. However, the precise mechanisms underlying these observations are still not fully clear, especially for specific populations of cells within the spinal cord.

Several key signalling pathways have been identified as major contributors to spinal cord I/R injury. Of these, the inflammatory pathway mediated via toll-like receptor 4 (TLR4) appears to be a key line of enquiry. TLR4 is known to initiate innate immune responses by sensing injury-induced endogenous ligands from necrotic cells such as heat-shock protein (HSP) and diverse microbial products such as lipopolysaccharide (LPS). TLR4 activation triggers signal transduction cascades mediated by the transcription factor nuclear factor κB (NF-κB) that drive gene expression of pro-inflammatory cytokines such as interleukin 1β (IL-1β), interleukin 6 (IL-6) and tumour necrosis factor α (TNF-α), which result in the evolution of neuronal damage and exacerbate inflammatory reactions [18, 19]. TLR4 are richly expressed on the cell membranes of microglia [the principle immune cells of the central nervous system (CNS)], and it has been postulated that microglial activation participates in I/R injury through the release of growth factors, chemokines, regulatory cytokines, and other toxic mediators [18, 20].

It has been shown that anaesthetic agents can modulate both the TLR4-mediated NF-κB signalling pathway and microglial activation. Isoflurane (Iso) exerted neuroprotection by reducing the expression of the TLR4-mediated NF-κB signalling pathway and alleviating microglial activation after cerebral I/R injury in vitro and in vivo [21]. In addition, perioperative treatment with propofol (Pro) and Dex significantly suppressed cerebral I/R injury and upregulation of TNF-α and IL-1β in rats [22]. Thus, we hypothesised that there might be a link between Dex-induced spinal cord neuroprotection and both TLR4 activity and microglial activation.

Apoptosis is one of the major mechanisms that leads to neuronal cell death after spinal cord I/R. It has been shown that the expression of caspase-3, a major apoptosis effector molecule in both the intrinsic and extrinsic apoptosis pathways, increases significantly after spinal cord I/R injury [23]. Treatment with a Pro-Dex combination has been found to inhibit apoptosis of cortical and hippocampus neurons caused by I/R injury, as evidenced by downregulation of caspase-3 [22]. We therefore postulated that the neuroprotective effects of Dex in spinal cord I/R injury may be due in part to inhibition of apoptosis.

In this study, we first examined the effects of Dex on hindlimb motor function and neuronal viability in a rabbit model of spinal cord I/R injury. Next, we evaluated the effects of the drug on spinal cord microglial activation, the TLR4-mediated NF-κB signalling pathway, and caspase-3 dependent apoptosis in spinal cord neurons.

## Methods

### Animals

The study (2016-029) was allowed by the Institutional Animal Care and Use Committee of Guangzhou Medical University, Guangzhou, PR China (Chairperson Lijun Dai) on 3 May 2016, and was performed in accordance with the Guide for the Care and Use of Laboratory Animals published by the US National Institutes of Health (NIH Publication No. 85-23, revised 1996).

Twenty-four healthy New Zealand male and female rabbits, weighing between 2.0 and 2.5 kg were divided randomly into three groups: Dex (spinal cord I/R injury + 10 µg/kg/h Dex intravenous infusion from before ischemia until reperfusion, *n *= 8), I/R (spinal cord I/R injury + 0.9% saline intravenous infusion for the same time, *n *= 8) and Sham (no spinal cord I/R injury or Dex + 0.9% saline intravenous infusion, *n *= 8).

The animals were kept at the temperature of 20–25 °C, and allowed ad libitum to feed and water during the presurgery and postsurgery periods. Before surgery all animals were fasted overnight with free access to water.

### Surgical procedures

Following cannulation of the left marginal ear vein, the rabbits were anesthetised with 10% chloral hydrate 2.5 ml/kg. Anaesthesia was maintained with 10% chloral hydrate intravenous infusion according to the vital signs and muscle relaxation of rabbits. Spontaneous ventilation was maintained with room air and the electric blanket was used to keep body temperature at 37 ± 0.2 °C. The left ear central artery blood pressure, heart rate, electrocardiogram, and oxygen saturation were measured during the surgery.

For rabbits in the I/R and Dex groups, a modified Zivin method (under sterile technique) was used to establish the model of spinal cord ischemia and reperfusion [24]. Firstly, the left femoral artery was catheterised to monitor the lower extremity blood pressure and estimate the effect of abdominal aorta blocking. Then an abdominal incision was made and the abdominal aorta was isolated at the level of the renal arteries, an arterial clip was placed on the abdominal aorta 0.5 cm distal to the left (lower) renal artery ostia, and the incision was temporarily closed. After 30 min, the incision was reopened, the clip was removed, and the incision was permanently closed with deep sutures and skin clips. The model success criteria were as follows: (1) after the abdominal aorta blocking, the femoral artery blood pressure waveform disappeared; (2) after reperfusion, the blood pressure waveform of the femoral artery recovered rapidly; (3) after the surgery, the rabbits could eat normally, the forelimb was normal, the hindlimb had a varying degree of activity disorder or paralysis. Animals in the Sham group underwent a similar surgical procedure but the abdominal aorta was not occluded. To prevent infection, all experimental animals were given an intramuscular injection of gentamicin 40,000 U after surgery.

### Drug administration

For animals in the Dex group, Dex was administered 30 min before the onset of ischemia, and continued until the reperfusion. Dex was infused into the left marginal ear vein at a dose of 10 µg/kg/h. For the I/R and Sham groups, the same volume of 0.9% saline was infused intravenously.

### Neurological assessment

The motor functions of the rabbits were assessed at 48 h after reperfusion by an independent observer according to the following Tarlov scoring system [25]: 0 = spastic paraplegia and no movement of the hindlimb; 1 = spastic paraplegia and slight movement of the hindlimb; 2 = good movement of the hindlimb but unable to stand; 3 = able to stand but unable to walk normally; and 4 = complete recovery and normal gait/hopping. Results were displayed as median (range).

After neurological evaluation, the rabbits in all groups were anaesthetised with 10% chloral hydrate 2.5 ml/kg and spinal cords from L2 to L6 were harvested for histological analysis. At the end of these procedures, all rabbits were sacrificed under deep anaesthesia.

### Measurement of TNF-α and IL-1β using ELISA

The tissue samples of spinal cords from L2 to L4 were homogenised in ice saline at the ratio of 1:9 in weight, then the supernatants were harvested by centrifugation at 3000 rpm for 20 min, and stored in liquid nitrogen for further study.

The concentrations of TNF-α and IL-1β in the spinal cord were measured by ELISA using monoclonal antibodies and the procedure recommended by the supplier (Shanghai Bairui Bioengineering Institute, China). The absorbance at 450 nm was determined using a microplate reader (PerkinElmer, USA). The concentration of TNF-α and IL-1β were calculated based on the standard curve and expressed in pg/mg protein of sample.

### Histological study

The tissue samples of spinal cords from L5 to L6 were firstly fixed in 10% buffered formalin for 48 h at room temperature, then embedded in paraffin and serially cut at 5 µm intervals for further study.

The paraffin-embedded sections were stained with haematoxylin–eosin (HE) according to the procedure recommended by the supplier (Solarbio, China), and images were captured using a Leica DMi8 inverted fluorescence microscope (Leica Microsystems, German). In cases in which the cytoplasm was diffusely eosinophilic, the motor neuron cells were considered to be necrotic or dead. When basophilic substance was seen, the motor neuron cells were considered to be viable or alive. The surviving intact motor neurons in the ventral grey matter were counted and calculated as average numbers per region/animal in a blind manner.

### Iba-1 immunoreactivity

Activated microglia cells demonstrate an increased expression of new markers including ionised calcium–binding adaptor molecule 1 (Iba-1). The fluorescence intensity of Iba-1 is therefore commonly used to quantify activated microglia cells [26].

Briefly, the 5 µm thick paraffin-embedded sections were firstly deparaffinised and blocked with 10% donkey serum albumin for 2 h at room temperature. Then the sections were incubated with a primary goat anti-Iba-1 antibody (1:250; ab5076, Abcam, UK) at 4 °C overnight. After incubation, the sections were rinsed with phosphate buffer saline (PBS) containing 0.1% Triton X-100 and incubated with Alexa 546–conjugated donkey anti-goat IgG antibody (1:1000; Thermo Fisher Scientific, USA) for 2 h at room temperature. Images were captured using a Leica DMi8 inverted fluorescence microscope (Leica Microsystems, German). The number of immunoreactive cells in the ventral grey matter were counted and calculated as average numbers per region/animal in a blind manner.

### TLR4 and NF-κB immunoreactivity

This immunoreactivity analysis was carried out to confirm the relationship with the TLR4-mediated NF-κB signalling pathway and the neuroprotection of Dex against spinal cord I/R injury.

As for TLR4 immunoreactivity detection, the 5 µm thick paraffin-embedded sections were stained with a primary mouse anti-TLR4 antibody (1:100; ab22048, Abcam, UK) at 4 °C overnight. After incubation with peroxidase-conjugated donkey anti-mouse IgG antibody (1:1000; Abcam, UK) for 2 h at room temperature, sections were developed using diaminobenzidine (DAB; Vector Laboratories, USA) and counterstained with haematoxylin (Solarbio, China).

As for NF-κB immunoreactivity detection, the primary antibody was mouse anti-NF-κB p65 antibody (1:250; MAB3026, Millipore, USA), and the secondary antibody was Alexa 488–conjugated donkey anti-mouse IgG antibody (1:1000; Thermo Fisher Scientific, USA).

Images were captured using a Leica DMi8 inverted fluorescence microscope (Leica Microsystems, German). The number of immunoreactive cells in the ventral grey matter were counted and calculated as average numbers per region/animal in a blind manner.

### TUNEL assay

A TUNEL (terminal deoxynucleotidyl transferase-mediated dUTP nick-end labelling) assay was used to identify double stranded DNA fragmentation, characteristic of DNA degradation by apoptosis [25].

A one-step TUNEL apoptosis assay kit was used according to the procedure recommended by the supplier (Beyotime Biotechnology, China). Briefly, the 5 µm thick paraffin-embedded sections were firstly deparaffinised and treated with proteinase K (20 µg/ml) for 15 min at room temperature. After rinsing with PBS, the sections were incubated with Alexa 488-conjugated TUNEL detection solution for 1 h at 37 °C. Images were photographed using a Leica DMi8 inverted fluorescence microscope (Leica Microsystems, German). Subsequently, the numbers of TUNEL positive cells in the ventral grey matter were counted and calculated as average numbers per region/animal in a blind manner.

### Caspase-3 immunoreactivity

The expression of caspase-3 in the ventral grey matter was also evaluated immunohistochemically. The method was the same as previously described. The primary antibody was goat anti-caspase-3 antibody (1:250; SC1225, Santa Cruz Biotechnology, USA), and the secondary antibody was Alexa 488–conjugated donkey anti-goat IgG antibody (1:1000; Thermo Fisher Scientific, USA). Images were photographed using a Leica DMi8 inverted fluorescence microscope (Leica Microsystems, German), and the number of immunoreactive cells in the ventral grey matter were counted and calculated as average numbers per region/animal in a blind manner.

### Statistical analysis

All statistical analyses were performed with SPSS version 20.0 software (SPSS Inc, USA). Measurement data were displayed as mean ± SEM and categorical variables were displayed as median (range). The significance of differences between the groups of continuous variables was assessed by one-way ANOVA followed by Tukey’s test for multiple comparisons. Physiologic parameters collected during the surgery were analyzed using repeated measures ANOVA with group (Sham, I/R and Dex) as the between-subject factor and timepoints as the within-subject factor. Categorical variables were processed with Kruskal–Wallis test followed by Nemenyi post hoc analysis. *P *< 0.05 was considered statistically significant.

## Results

### Physiologic Parameters

The left ear central artery mean arterial pressure (MAP) and heart rate (HR) were comparable throughout the experiments in all groups (Table 1). During surgery, the animals’ vital signs from each group were relatively stable. There was no difference in MAP and HR among all groups (*P* > 0.05).

**Table 1:** The left ear central artery mean arterial pressure and heart rate at different time points from each group

Group	Before ischemia (30 min)	Before ischemia (15 min)	At starting ischemia	After ischemia (15 min)	At starting reperfusion	After reperfusion (15 min)
MAP (mmHg)						
Sham	85.0 ± 3.5	82.0 ± 3.9	80.0 ± 2.8	78.0 ± 2.5	79.0 ± 3.2	83.0 ± 4.2
I/R	84.0 ± 4.2	83.0 ± 2.5	78.0 ± 3.5	76.0 ± 3.2	79.0 ± 1.8	82.0 ± 2.5
Dex	84.0 ± 4.2	80.0 ± 2.8	74.0 ± 2.5	76.0 ± 2.8	79.0 ± 1.8	82.0 ± 2.5
HR (beats/min)						
Sham	258.0 ± 7.8	250.0 ± 6.4	246.0 ± 5.3	247.0 ± 4.9	253.0 ± 6.7	255.0 ± 6.4
I/R	252.0 ± 6.7	248.0 ± 8.1	242.0 ± 6.4	240.0 ± 3.5	249.0 ± 6.0	253.0 ± 4.2
Dex	260.0 ± 6.0	251.0 ± 8.1	234.0 ± 4.9	239.0 ± 3.5	244.0 ± 4.2	246.0 ± 3.2

Data are presented as mean ± SEM (*n* = 8)

*MAP* mean arterial pressure, *HR* heart rate

### Dex treatment preserved neurological assessment scores after spinal cord I/R injury

**Fig. 1:**
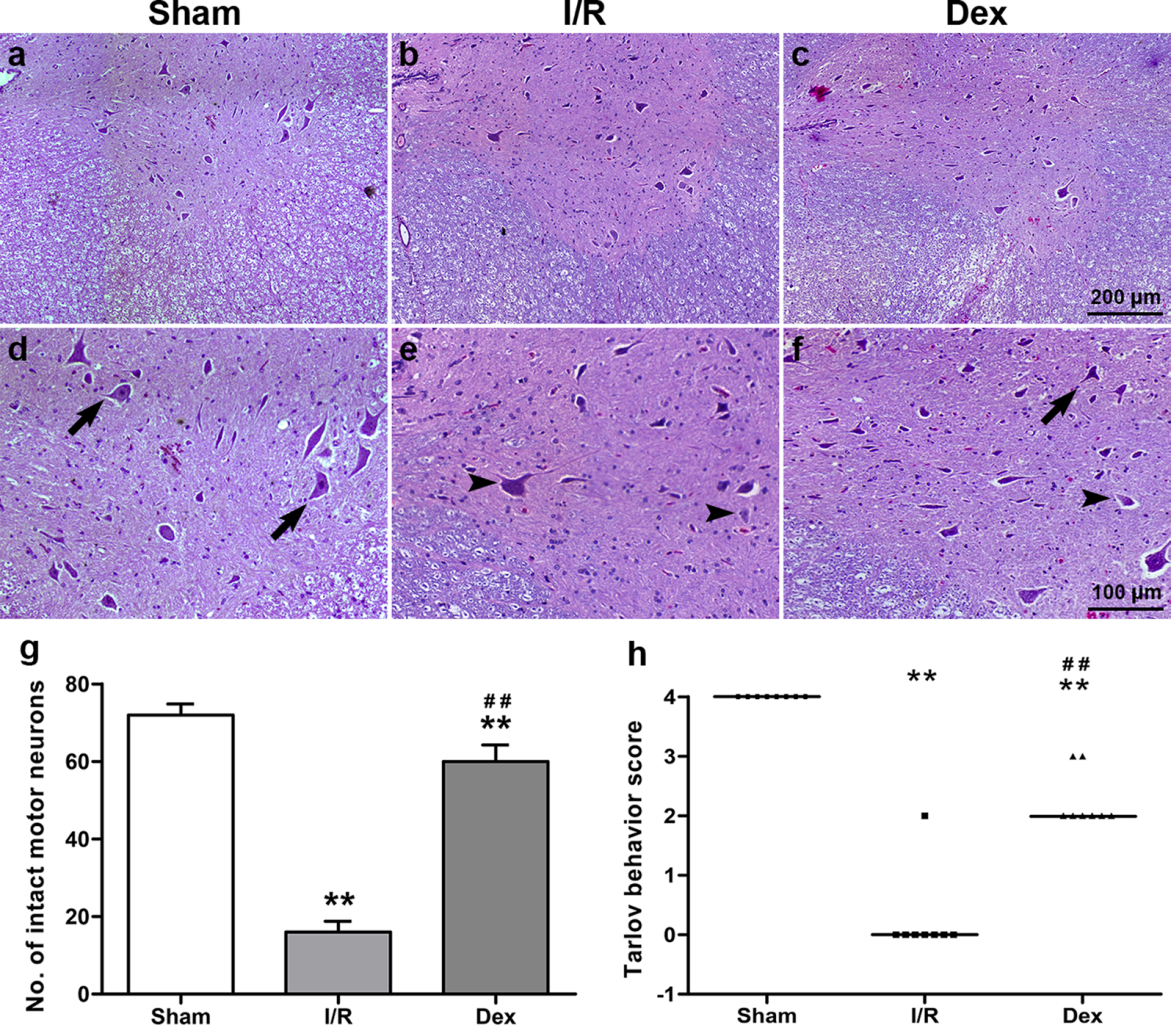
The neurological motor function and histological assessment of the spinal cord at 48 h after reperfusion from each group. **a**–**f** Representative sections of ventral grey matter stained with haematoxylin-eosin. Arrows indicate the viable or alive motor neurons and arrowheads indicate the necrotic or dead motor neurons. Upper scale bar = 200 μm, lower scale bar = 100 μm. **g** Number of intact motor neurons in the ventral grey matter. Data are presented as mean ± SEM (*n *= 8). **h** The Tarlov behaviour scores at 48 h after reperfusion from each group, each symbol represents data for one rabbit, bar = median (*n *= 8). **P < 0.05 versus Sham; ^##^*P * < 0.05 versus I/R

The individual neurological scores of the three groups after surgery are shown in Fig. 1h. Animals in the Sham group had normal neurological outcome [median Tarlov score 4 (4, 4)]. After 48 h of spinal cord I/R injury, all animals in the I/R and Dex groups displayed varying degrees of paraparesis; the severity of motor dysfunction was significantly lower in the Dex group [median Tarlov score 0 (0, 2) in the I/R group and 2 (2, 3) in the Dex group; *P *< 0.05].

### Dex treatment preserved histological assessment scores after spinal cord I/R injury

The histological changes of the three groups after surgery are shown in Fig. 1a–g. In the Sham group, positive cells in the HE-stained sections were abundantly detected. Compared with the Sham group, the I/R group demonstrated significant loss of motor neurons (*P *< 0.05). Dex treatment resulted in significant protective effects on neuron survival following spinal cord I/R injury (*P *< 0.05).

### Dex treatment decreased microglial activation after spinal cord I/R injury

**Fig. 2:**
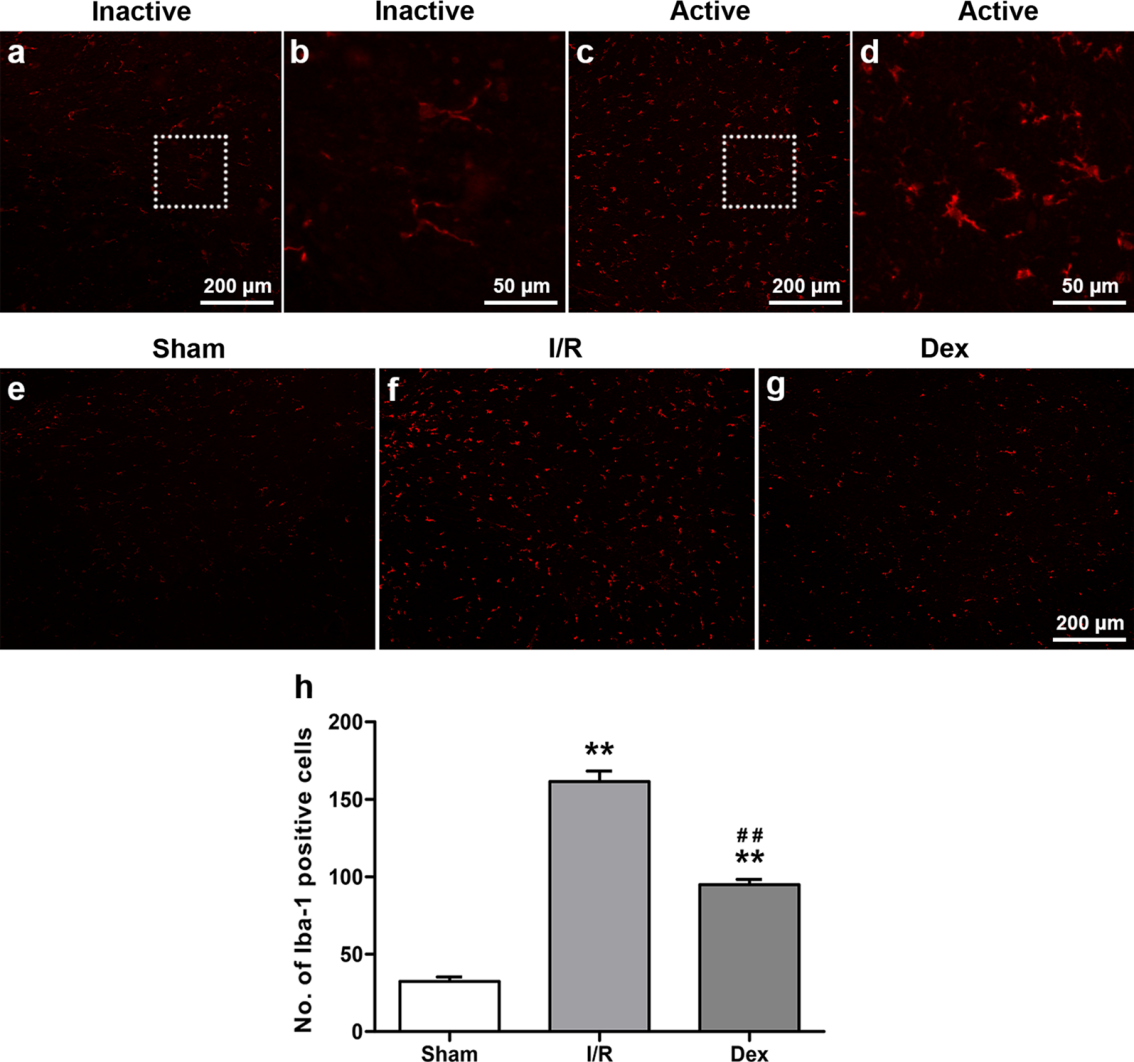
The microglial reaction at 48 h after reperfusion from each group. **a**–**d** Effects on the morphology changed in microglia cells after spinal cord I/R injury. In **a**, **c**, scale bar = 200 μm; in **b**, **d**, scale bar = 50 μm. **e**–**g** Effects on immunoreactivity to Iba-1 in the ventral grey matter. Scale bar = 200 μm. **h** Number of Iba-1 positive cells in the ventral grey matter. Data are presented as mean ± SEM (*n* = 8). **P < 0.05 versus Sham; ^##^*P* < 0.05 versus I/R

As microglial cells are activated, the cells undergo morphological changes in which the cell body becomes relatively large and the processes become shorter [27, 28]. As shown in Fig. 2a–d, activated microglia cells with increased Iba-1 fluorescence exhibited these morphological changes and could easily be distinguished from the inactivated ones. As shown in Fig. 2e–h, increased immunoreactivity of Iba-1 was observed in the I/R group in comparison to the Sham group (*P *< 0.05). In rabbits treated with Dex, this increase in Iba-1 immunoreactivity in the ventral grey matter was significantly inhibited indicating decreased microglial activation (*P * < 0.05).

### Dex treatment inhibited the TLR4-mediated NF-κB signalling pathway after spinal cord I/R injury

**Fig. 3:**
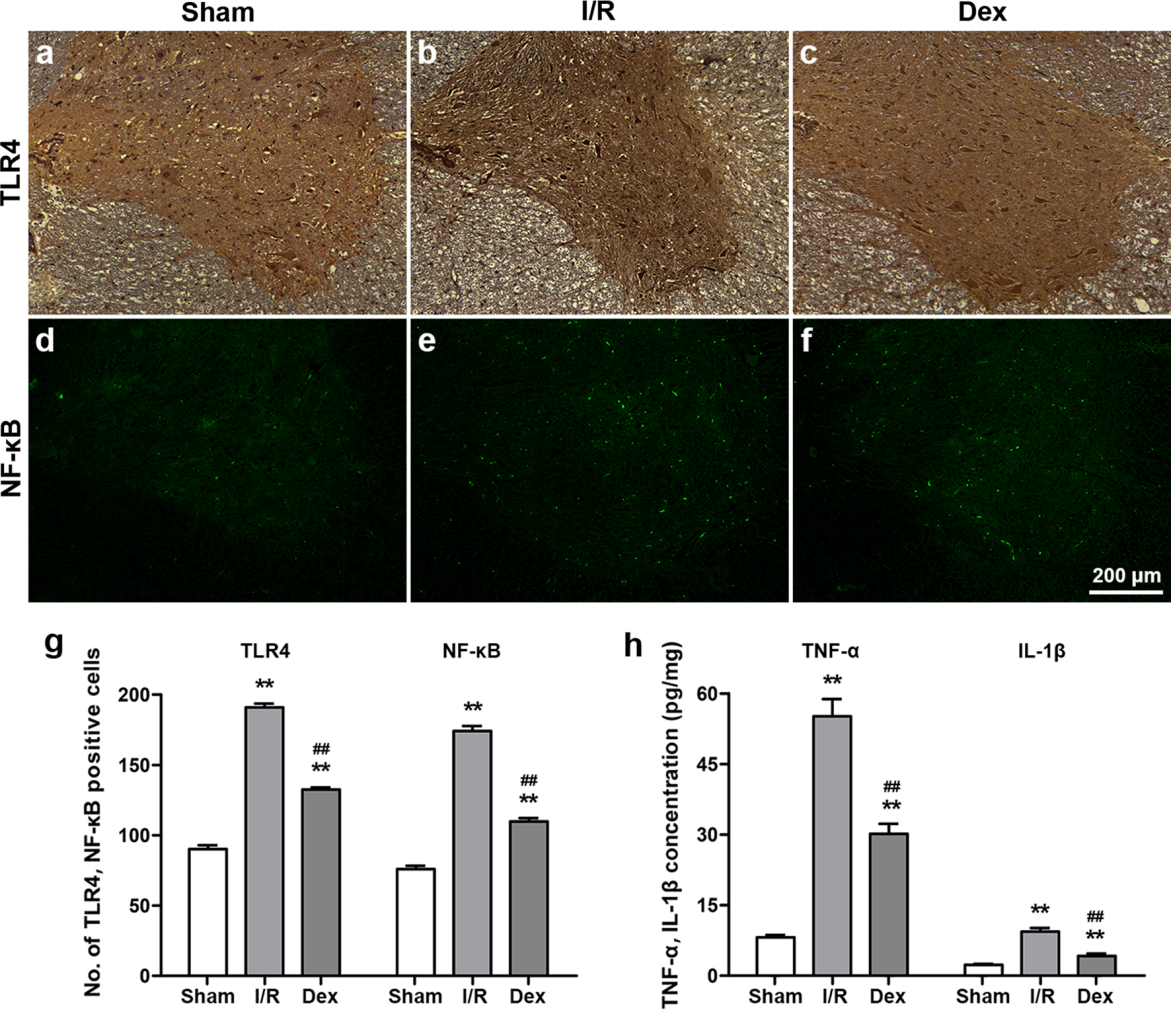
The TLR4-mediated NF-κB signalling pathway at 48 h after reperfusion from each group. **a**–**c** Effects on immunoreactivity to TLR4 in the ventral grey matter, which used DAB staining counterstained with haematoxylin. **d**–**f** Effects on immunoreactivity to NF-κB in the ventral grey matter. **g** Number of TLR4 and NF-κB positive cells in the ventral grey matter. **h** The concentration of TNF-α and IL-1β in the spinal cord, as assessed by ELISA. All data are presented as mean ± SEM (*n *= 8). Scale bar = 200 μm. **P < 0.05 versus Sham; ^##^*P * < 0.05 versus I/R

The expression of TLR4 and NF-κB was measured to further investigate its effect on microglial activation during I/R-induced inflammatory processes. As shown in Fig. 3a–g, the I/R group markedly increased immunoreactivity of TLR4 and NF-κB in comparison to the Sham group at 48 h after reperfusion (*P *< 0.05). Dex treatment inhibited TLR4 and NF-κB immunoreactivity following spinal cord I/R injury (*P *< 0.05).

Furthermore, to verify the inflammatory signalling pathway downstream activation, we detected the pro-inflammatory cytokines TNF-α and IL-1β activation by ELISA. As shown in Fig. 3h, the data presented that these cytokines were activated along with the activation of TLR4 and NF-κB (*P *< 0.05).

### Dex treatment inhibited caspase-3 dependent apoptosis after spinal cord I/R injury

**Fig. 4:**
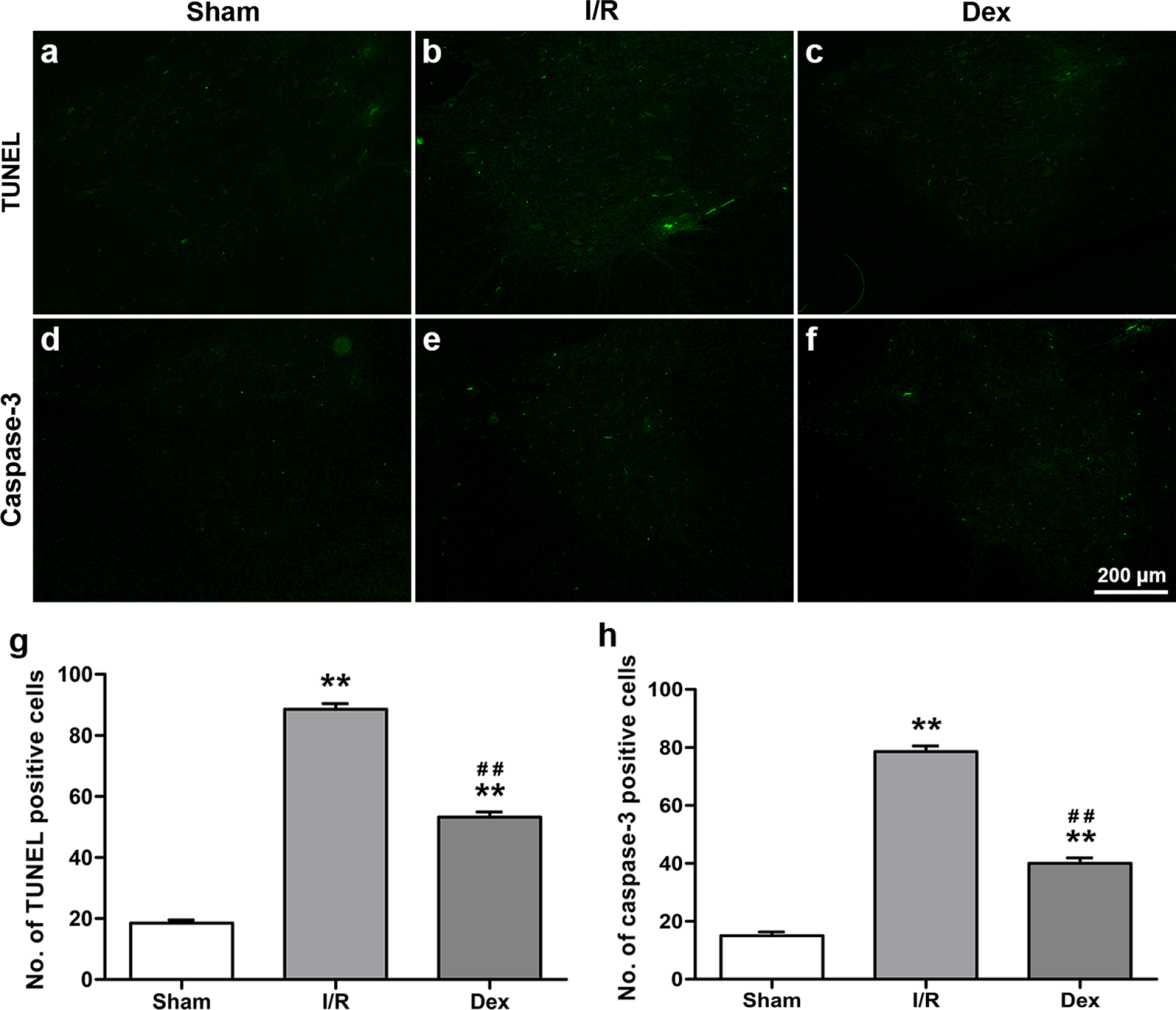
The caspase-3 dependent apoptosis at 48 h after reperfusion from each group. **a**–**c** Effects on TUNEL staining in the ventral grey matter. **d**–**f** Effects on immunoreactivity to caspase-3 in the ventral grey matter. **g** Number of TUNEL positive cells in the ventral grey matter. **h** Number of caspase-3 positive cells in the ventral grey matter. All data are presented as mean ± SEM (*n *= 8). Scale bar = 200 μm. **P <0.05 versus Sham; ^##^*P* < 0.05 versus I/R

To observe a possible relationship between caspase-3 dependent apoptosis and the neuroprotective effects of Dex, TUNEL staining and caspase-3 immunoreactivity analysis were performed. As shown in Fig. 4, there were few TUNEL positive cells in the Sham group, while numerous TUNEL positive cells were observed in the I/R group (*P *< 0.05). On the other hand, Dex treatment significantly reduced the number of apoptotic cells (*P* < 0.05). The caspase-3 immunoreactivity analysis was similar (*P* < 0.05).

## Discussion

Patients undergoing aortic surgery remain at high risk of perioperative complications despite recent surgical advances in the field. Many of these potential complications are a consequence of tissue ischemia and reperfusion resulting from disruption of normal blood flow in the aorta with subsequent free radical formation, mitochondrial failure, and release of pro-inflammatory cytokines. Spinal cord I/R injury is a serious complication of major aortic surgery and can lead to severe and long-term disability. Measures that address this devastating condition are therefore of great interest. The pathophysiology of neuronal loss in spinal cord I/R injury is complex and unravelling the underlying processes is key in developing novel neuroprotective therapies [29].

The protective effects of Dex in a wide range of organ systems have been well documented in preclinical studies [6–15] and the drug has already shown promise in improving neuronal viability in animal models of spinal cord I/R injury [16, 17]. Clinical trials have also demonstrated beneficial effects of Dex in reducing both biomarkers of organ damage and clinical outcomes following I/R injury in other systems [30, 31]. Our initial results confirm previous observations that Dex preserves hindlimb motor function and reduces neuronal loss following aortic cross clamping in a rabbit model of spinal cord I/R injury [16, 17]. The main aim of this study was therefore to investigate the mechanisms underlying these neuroprotective effects in spinal cord I/R injury with a focus on inflammation and cell death pathways.

The interaction between microglia and neurons in the pathophysiology of diseases of the CNS is an area of great interest [32, 33]. Microglia are macrophage-like cells that act as the main immune cells of the CNS. They are responsible for defence against infectious agents as well as removing plaques and damaged cells. One of the key signalling pathways that has been identified between injured neurons and microglia is mediated via TLR4. The activation of TLR4 on microglial cell membranes by injury-induced ligands activates the transcription factor NF-κB which in turn leads to the upregulation and release of a number of pro-inflammatory cytokines with exacerbation of damage to the injured neurons. This pathway has been implicated in a number of neurodegenerative diseases as well as in spinal cord I/R [18, 19, 34, 35]. Inhibition of the TLR4-mediated NF-κB signalling pathway has been strongly implicated as one of the mechanisms underlying the protective effects of Dex in a number of other organ systems [11, 36] and this led us to hypothesise that it may be one of the ways in which Dex ameliorates the effects of spinal cord I/R injury. Our results clearly show that Dex treatment decreases microglia activation following spinal cord I/R injury. We also show that Dex ameliorates the upregulation of TLR4 and NF-κB immunoreactivity in the ventral horn following spinal cord I/R injury and reduces the release of the pro-inflammatory cytokines TNF-α and IL-1β (known downstream effector molecules in the TLR4/NF-κB pathway). While we have demonstrated in this current study that Dex has significant effects on these two processes, our results do not conclusively demonstrate a causal relationship between microglial activation after spinal cord injury and the observed upregulation of TLR4 and NF-κB; this is an area to explore in more details in our future work. Taken together, these data strongly suggest that the neuroprotective effects of Dex seen in this model are mediated, at least in part, through a reduction in spinal cord inflammation and an improvement in metabolic tolerance due to downregulation of these pathways.

Studying the effects of a novel therapy on neuronal cell death pathways is also crucial in understanding its neuroprotective effects. Apoptosis is a major form of regulated cell death that operates via mitochondria dependent and independent signalling pathways with the caspase family of cysteine proteases playing an essential role in both pathways. Caspase-3, which is one of the most important apoptosis effector molecules, is necessary for the cleavage of a large number of proteins and for apoptosis-associated chromatin margination, DNA fragmentation, and nuclear collapse. As such it can be a useful marker of apoptosis. Here we show that both the increase in number of neurons undergoing apoptosis and the increase in caspase-3 expression following spinal cord I/R injury are significantly reduced by Dex treatment. Whether this is due to direct effects on key mediators in the apoptosis cell signalling pathways or whether it reflects a more indirect process (e.g. less regulated neuronal death as a result of reduced spinal cord inflammation following Dex treatment) is not clear from this study.

**Fig. 5:**
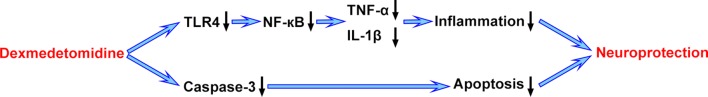
The model depicting the mechanism by which Dex treatment exerted neuroprotective effects by regulation of the TLR4-mediated NF-κB inflammatory system and the caspase-3 mediated neuronal apoptosis system after spinal cord I/R injury

Taken together, our data provide evidence that Dex may be a potential neuroprotective therapeutic agent for spinal cord I/R injury. We have also shown that this is due (at least in part) to the downregulation of two important parallel pathways that reduce neuronal viability, namely the TLR4-mediated NF-κB inflammatory system and the caspase-3 mediated system of neuronal apoptosis (Fig. 5). A suppression of microglial activation also appears to play a role. However, this is unlikely to be the whole story and future work should focus on further elucidating the mechanisms of Dex treatment in spinal I/R injury. The effect on other neuronal cell death pathways (both regulated and unregulated) would be particularly interesting.

A number of clinical trials have demonstrated that Dex has significant therapeutic benefits over other anaesthetic and sedative agents in terms of reducing neurological complications such as postoperative delirium [37], and other trials have concluded that it shows promise in reducing organ injury in a range of other systems [30, 31, 38]. All of these trials have also confirmed that Dex is a relatively safe agent with a wide therapeutic window (the most common complications of hypotension and bradycardia can usually be managed easily). A potential limitation of our study is that the dose of Dex used in the treatment group was higher than that currently licenced for anaesthetic/sedative use in humans; this is an issue that would need to be addressed before appropriate clinical trials could take place. However, given the established safety profile of the drug at lower doses and the neuroprotective effects demonstrated in preclinical studies, randomised clinical trials assessing the efficacy of this promising therapy in the treatment of spinal cord I/R injury would be timely.

## Conclusions

In Summary, we found that Dex confers neuroprotection against spinal cord I/R injury through suppression of spinal cord inflammation and neuronal apoptosis. Suppression of microglial activation and inhibition of both the TLR4-mediated NF-κB signalling pathway and caspase-3 dependent apoptosis are implicated.

## Abbreviations

Dex: dexmedetomidine
I/R: ischemia–reperfusion
HE: haematoxylin–eosin
TUNEL: terminal deoxynucleotidyl transferase-mediated dUTP biotin nick end labelling
Iba-1: ionised calcium-binding adaptor molecule 1
TLR4: toll-like receptor 4
NF-κB: nuclear factor κB
IL-1β: interleukin 1β
TNF-α: tumour necrosis factor α
ELISA: enzyme-linked immunosorbent assays
HSP: heat-shock protein
LPS: lipopolysaccharide
IL-6: interleukin 6
CNS: central nervous system
Iso: isoflurane
Pro: propofol
PBS: phosphate buffer saline
MAP: mean arterial pressure
HR: heart rate
